# Comparative volumetric wear of occlusal splint materials under simulated gastric acid and artificial saliva conditions

**DOI:** 10.1186/s12903-026-10055-5

**Published:** 2026-10-01

**Authors:** Kübra Yıldız Domaniç, Begüm Giritlioğlu Öztürk, Muzaffer Ateş

**Affiliations:** 1 Department of Prosthodontics, Faculty of Dentistry, Istanbul Atlas University, Istanbul, Türkiye; 2 Department of Prosthodontics, Faculty of Dentistry, Istanbul Medipol University, Istanbul, Türkiye

**Keywords:** Occlusal splint, Volumetric wear, PEEK, CAD/CAM, 3D printing, Simulated gastric acid

## Abstract

**Background:**

Wear of occlusal splint materials may alter appliance geometry and occlusal relationships during use. This in vitro study evaluated the volumetric material loss of four commercially available occlusal splint materials differing in polymer composition and manufacturing technology after mechanical loading in artificial saliva and simulated gastric acid.

**Methods:**

A total of 128 specimens were prepared from two digital light processing (DLP)-printed PMMA-based resins (Detax Freeprint Splint and Armarez), one CAD/CAM-milled PEEK material (PEEK Biosolution), and one conventionally injection-molded polyamide material (Cool&Flex). The specimens were allocated to artificial saliva or simulated gastric acid and subjected to either 30,000 or 120,000 chewing cycles. Baseline and post-simulation stereolithography datasets were superimposed, and volumetric material loss was calculated in mm³. The effects of material, storage medium, chewing-cycle number, and their interactions were evaluated using robust factorial ANOVA based on 20% trimmed means.

**Results:**

Volumetric material loss was significantly associated with material (*p* < 0.001, partial η² = 0.881), chewing-cycle number (*p* = 0.001, partial η² = 0.626), and the material × chewing-cycle interaction (*p* = 0.001, partial η² = 0.752). The main effect of storage medium was not statistically significant (*p* = 0.054). When values were pooled across both storage media and chewing-cycle conditions, Cool&Flex showed the greatest volumetric material loss (20% trimmed mean ± SE: 1.171 ± 0.193 mm³), whereas comparatively low values were observed for PEEK Biosolution (0.079 ± 0.014 mm³) and Armarez (0.087 ± 0.020 mm³).

**Conclusions:**

Under the conditions of this in vitro study, volumetric material loss differed among the tested commercial products and increased with mechanical loading, although the magnitude of this increase varied by material. Storage in simulated gastric acid did not significantly affect volumetric material loss. Because polymer composition and manufacturing method varied simultaneously among the products, their independent effects cannot be distinguished. These findings are limited to the materials and experimental conditions evaluated and should not be interpreted as direct predictions of clinical longevity.

## Introduction

Occlusal splints are widely used in the management of bruxism, temporomandibular disorders (TMDs), and other parafunctional conditions. Their primary functions include protecting dental structures, redistributing occlusal forces, reducing muscular hyperactivity, and minimizing excessive loading of the temporomandibular joint. The long-term clinical effectiveness of these appliances depends not only on their design but also on the physical, mechanical, and tribological properties of the materials used for their fabrication. Among these properties, wear resistance is particularly important because material degradation may alter occlusal relationships, compromise treatment outcomes, reduce appliance longevity, and ultimately necessitate replacement [1–3].

Recent advances in digital dentistry have considerably expanded the range of materials and manufacturing technologies available for occlusal splint fabrication. Conventional polymer-based materials are increasingly being supplemented or replaced by CAD/CAM-milled polymers and 3D-printed photopolymer resins, offering improved manufacturing precision, reproducibility, and workflow efficiency. However, manufacturing technology also influences polymerization efficiency, degree of conversion, residual monomer content, internal porosity, surface characteristics, and mechanical behavior, all of which may affect the long-term wear performance of splint materials. High-performance polymers such as CAD/CAM-milled polyetheretherketone (PEEK), DLP 3D-printed polymethyl methacrylate (PMMA)-based resins, and conventionally processed polyamide materials have therefore attracted considerable attention because of their favorable mechanical properties, biocompatibility, and clinical applicability [4–7].

In addition to repeated mechanical loading during mastication and parafunctional activities, dental polymers are continuously exposed to complex chemical challenges within the oral environment. Variations in salivary composition, temperature, pH, dietary acids, and intrinsic acids may contribute to progressive material degradation. Among these factors, gastric acid exposure associated with gastroesophageal reflux disease (GERD), chronic vomiting, or other gastrointestinal disorders represents one of the most aggressive acidic conditions encountered in the oral cavity. Previous studies have demonstrated that acidic environments may adversely affect dental polymers by increasing surface roughness, reducing hardness, accelerating hydrolytic degradation, and compromising structural integrity [8–10]. Nevertheless, the influence of gastric acid on the volumetric wear behavior of contemporary occlusal splint materials, particularly those produced using different manufacturing technologies, remains insufficiently understood.

Previous investigations have compared the wear behavior of conventionally processed, CAD/CAM-milled, and additively manufactured occlusal splint materials under simulated mastication [11–14]. These studies consistently demonstrated that industrially polymerized CAD/CAM materials generally exhibit superior wear resistance owing to their higher degree of conversion, lower residual monomer content, and reduced internal porosity. More recently, Lawson et al. reported that the wear resistance of commercially available 3D-printed occlusal device materials differed substantially according to resin formulation, highlighting that material composition may be as influential as the manufacturing process itself [15]. Likewise, Aminuddin and Petridis demonstrated significant differences in flexural strength, monomer release, and wear among conventionally fabricated, milled, and 3D-printed occlusal splint materials, confirming that manufacturing technique directly affects the long-term mechanical performance of these polymers [16]. Furthermore, Arreaza et al. compared the wear behavior of occlusal device materials manufactured using different fabrication techniques and reported significant differences in wear resistance between conventionally processed, CAD/CAM-milled, and additively manufactured materials, emphasizing the importance of both polymer composition and processing method in determining clinical durability [17].

Despite these important advances, previous studies have largely evaluated wear under neutral laboratory conditions or focused on individual fabrication techniques. To date, limited evidence is available regarding the combined effects of cyclic mechanical loading and aggressive acidic aging across commercially available occlusal splint materials manufactured using different technologies. Consequently, the relative contributions of polymer composition, manufacturing technology, and acidic aging to volumetric material loss remain unclear.

Based on the available evidence, we hypothesized that the CAD/CAM-milled PEEK material would demonstrate the lowest volumetric wear because of its semi-crystalline structure, high degree of industrial polymerization, and excellent mechanical stability, whereas the conventionally injection-molded polyamide material (Cool&Flex) would exhibit the greatest wear because of its comparatively lower hardness, greater water sorption, and increased susceptibility to plastic deformation. Furthermore, although the two investigated DLP 3D-printed PMMA-based materials were fabricated using the same manufacturing technology, differences in resin formulation and oligomer composition were expected to result in different wear behaviors, thereby enabling assessment of whether material composition or manufacturing technology exerts the greater influence on wear resistance. In addition, because acidic environments have been reported to differentially affect the surface hardness and roughness of dental polymers according to their chemical composition [9, 10], a possible interaction between storage medium and material type under simulated gastric acid exposure was anticipated.

Unlike previous investigations, the present study simultaneously compares four commercially available occlusal splint materials representing three different manufacturing technologies under both artificial saliva and simulated gastric acid conditions using three-dimensional volumetric wear analysis. This design enables both within-technology (between two DLP 3D-printed PMMA-based resins) and between-technology comparisons under identical experimental conditions, thereby providing a more comprehensive evaluation of the relative influence of material composition and manufacturing technology on wear resistance.

To the best of our knowledge, this is the first study to simultaneously evaluate four commercially available occlusal splint materials fabricated using three distinct manufacturing technologies: two DLP 3D-printed PMMA-based resins (Detax Freeprint Splint and Armarez), one CAD/CAM-milled PEEK material (PEEK Biosolution), and one conventionally injection-molded polyamide material (Cool&Flex). All specimens were fabricated by the same dental laboratory to minimize manufacturing variability and were assessed under two clinically relevant chewing-cycle protocols and two storage media representing physiological (artificial saliva) and pathological (simulated gastric acid) conditions using three-dimensional volumetric superimposition.

Therefore, the aim of this in vitro study was to evaluate the volumetric wear behavior of four commercially available occlusal splint materials representing different manufacturing technologies following simulated mastication in artificial saliva and gastric acid environments. Volumetric material loss was quantified using three-dimensional digital superimposition of pre- and post-simulation scans. The null hypothesis was that material type, storage medium, and chewing cycle number would not significantly influence volumetric material loss.

## Materials and methods

### Study design

This in vitro experimental study evaluated the volumetric wear behavior of four commercially available occlusal splint materials differing in polymer composition and manufacturing technology. The materials included two polymethyl methacrylate (PMMA)-based resins produced by digital light processing (DLP), one CAD/CAM-milled polyetheretherketone (PEEK) material, and one conventionally injection-molded polyamide material.

The experimental design included two storage media, artificial saliva and simulated gastric acid, representing neutral and acidic environmental conditions, respectively, and two mechanical loading protocols comprising 30,000 and 120,000 chewing cycles. Based on the commonly cited approximation that 240,000 chewing cycles represent approximately one year of clinical function, these protocols were selected to represent approximately 1.5 and 6 months of simulated functional loading, respectively [6, 7]. These two cycle levels also allowed the wear behavior of the materials to be compared after shorter and more extended periods of mechanical loading. The factorial design enabled evaluation of the main effects of material, storage medium, and chewing-cycle number, as well as their interactions, on volumetric material loss.

A total of 128 standardized specimens were fabricated and equally allocated to the experimental groups. Each specimen was digitally scanned before mechanical loading to obtain a baseline stereolithography (STL) dataset. After completion of the assigned chewing-simulation protocol, all specimens were rescanned using the same scanning procedure. Volumetric material loss was subsequently determined by three-dimensional superimposition of the baseline and post-simulation STL datasets.

### Materials

Four commercially available occlusal splint materials manufactured using three different manufacturing technologies were included in the study. These consisted of two DLP 3D-printed PMMA-based resins (Detax Freeprint Splint and Armarez), one CAD/CAM-milled PEEK material (PEEK Biosolution), and one conventionally injection-molded polyamide material (Cool&Flex). To minimize manufacturing-related variability, all specimens were fabricated by the same dental laboratory (Armaera Dental Laboratory, Istanbul, Türkiye) following standardized production protocols (Table1).

**Table 1:** Occlusal splint materials investigated in the study

Group	Material	Polymer composition		Manufacturing technology	Manufacturer	*n*
G1	Detax Freeprint Splint	PMMA-based photopolymer resin		DLP 3D printing	DETAX GmbH, Ettlingen, Germany	32
G2	Armarez	PMMA-based photopolymer resin		DLP 3D printing	Armaera Dental Laboratory, Istanbul, Türkiye	32
G3	PEEK Biosolution	Polyetheretherketone (PEEK)		CAD/CAM milling	Merz Dental GmbH, Lütjenburg, Germany	32
G4	Cool&Flex	Polyamide		Conventional injection molding	Cooldent, Balıkesir, Türkiye	32
Total						128

### Specimen preparation

All specimens were prepared as standardized cylindrical discs measuring 16 mm in diameter and 3 mm in thickness.

The Detax Freeprint Splint and Armarez specimens were designed using Exocad software (exocad GmbH, Darmstadt, Germany) and fabricated using digital light processing (DLP) technology on an Asiga MAX 2 DLP 3D printer (Asiga, Sydney, Australia). Printing was performed using a vertical build orientation (90°) with a layer thickness of 100 μm in accordance with the manufacturers’ recommended printing parameters. Build-file preparation required approximately 7–11 min per print job. Following printing, uncured resin was removed by ultrasonic cleaning in 91% isopropyl alcohol for 7–8 min. Final post-curing was subsequently performed using a Dentmate ProMax BL curing unit (Dentmate Technology Co. Ltd., Taiwan) according to the manufacturers’ recommended post-polymerization protocols to achieve complete polymerization prior to testing. After post-curing, the specimens were inspected for residual support material, and any remaining support attachment areas were carefully removed according to the manufacturers’ recommended finishing procedures to obtain standardized test surfaces.

PEEK Biosolution specimens were fabricated from pre-polymerized CAD/CAM blocks using a five-axis milling machine. Milling was performed using a single-flute carbide bur under controlled cutting conditions to minimize heat generation, internal stresses, and potential surface damage. Following milling, the specimens were sequentially finished using 400-, 800-, 1200-, and 1500-grit silicon carbide abrasive papers under continuous water cooling. Pre-polishing was performed using reinforced rubber polishers, followed by final polishing with a universal polishing paste according to the manufacturer’s recommendations.

Cool&Flex specimens were fabricated using a conventional injection-molding technique according to the manufacturer’s instructions. Following deflasking, excess material and flashes were removed in accordance with the manufacturer’s standard laboratory finishing protocol to obtain standardized specimen surfaces prior to testing.

After fabrication, all specimens were embedded in autopolymerizing acrylic resin blocks to ensure reproducible positioning during digital scanning and chewing simulation. Three reference notches were created on the peripheral acrylic surfaces of each specimen holder. These reference landmarks were used exclusively for geometric registration during digital superimposition and were excluded from the wear measurement area. 

### Experimental groups

Each material group consisted of 32 specimens, which were randomly allocated into four experimental subgroups according to storage medium and chewing cycle number, resulting in eight specimens per subgroup (Table 2). 

**Table 2:** Experimental groups

Subgroup	Storage medium	Chewing cycles	*n*
S1	Artificial saliva	30,000	8
S2	Simulated gastric acid	30,000	8
S3	Artificial saliva	120,000	8
S4	Simulated gastric acid	120,000	8

Overall experimental design:

$$\begin{aligned}\text{4 materials}\times\text{2 storage media}&\\\times\text{2 chewing cycle protocols}\\\times\text{8 specimens}=\text{128 specimens}\end{aligned}$$

### Preparation of storage media

Two storage media were prepared: artificial saliva and simulated gastric acid. Artificial saliva was prepared using analytical-grade chemicals according to the formulation presented in (Tables 3 and 4). The components were dissolved in purified water and mixed until a homogeneous solution was obtained.

**Table 3:** Composition of artificial saliva

Component	Amount
NaCl	1.30 g
MgCl₂·6 H₂O	0.03 g
CaCl₂·2 H₂O	0.08 g
KCl	0.30 g
KOH	0.90 g
H₃PO₄	472 µL

**Table 4:** Composition of simulated gastric acid

Parameter	Value
Acid	Hydrochloric acid
Concentration	0.06 M
Solution concentration	0.113%
pH	1.2

Simulated gastric acid consisted of hydrochloric acid prepared at a concentration of 0.06 M, corresponding to 0.113% HCl, with a final pH of 1.2, thereby simulating the acidic conditions associated with gastric reflux.

### Initial digital scanning

Prior to chewing simulation, all specimens were scanned using a Shining 3D DS-MIX laboratory scanner (Shining 3D Tech Co., Ltd., Hangzhou, China) while maintaining identical positioning on their acrylic specimen holders. Baseline digital models were exported in stereolithography (STL) format for subsequent volumetric analysis.

The reference notches created on the acrylic specimen holders were intentionally included in every scan to ensure reproducible alignment during three-dimensional digital superimposition. These reference landmarks facilitated standardized geometric registration while remaining outside the wear measurement area.

### Chewing simulation

**Fig. 1:**
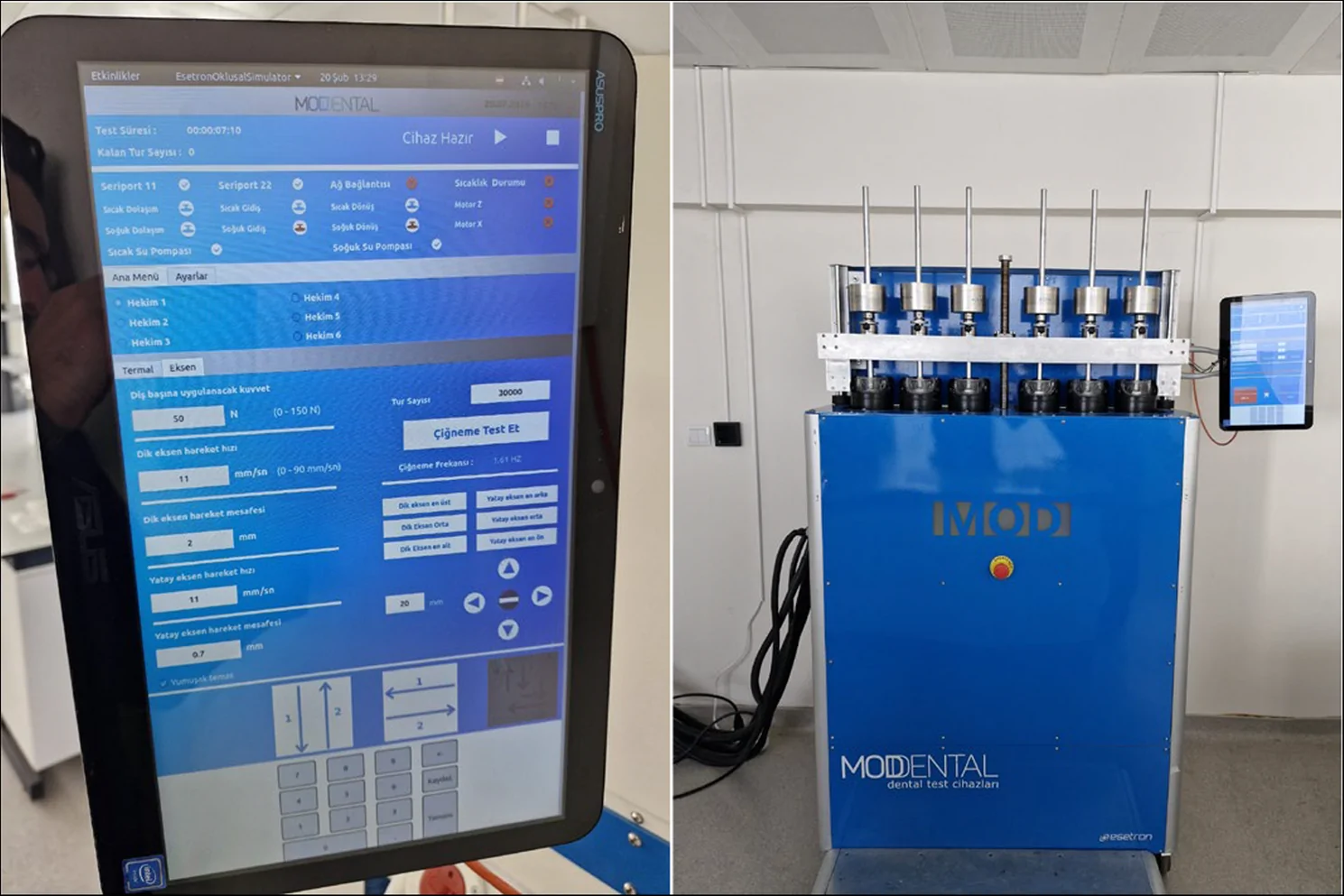
MODDENTAL/Esetron dual-axis chewing simulator used for standardized mechanical loading of the specimens

Mechanical loading was performed using a MODDENTAL/Esetron dual-axis chewing simulator (Esetron Semi İletken Bilgisayar Teknolojileri A.Ş., Ankara, Türkiye) at the Faculty of Dentistry, Atatürk University (Fig. 1). The device reproduced controlled vertical and horizontal movements under standardized loading conditions. A hemispherical stainless-steel antagonist with a diameter of 4 mm was used to produce a standardized two-body wear pattern.

Throughout mechanical loading, the specimens remained continuously immersed in their assigned medium, either artificial saliva or simulated gastric acid. Thus, chemical exposure and mechanical loading occurred simultaneously for the entire duration of the respective chewing-simulation protocol. The media were maintained at 37 °C throughout testing. Although the simulator was equipped with a thermocycling function, thermocycling was not used in this study.

A vertical load of 50 N was applied at a frequency of 1.61 Hz. The vertical movement distance was 2 mm, and the horizontal sliding distance was 0.7 mm. Vertical and horizontal movement speeds were both maintained at 11 mm/s. The specimens were subjected to either 30,000 or 120,000 chewing cycles, representing approximately 1.5 and 6 months of simulated functional loading, respectively, as described in “Study Design”. The chewing simulation parameters are summarized in Table 5. 

**Table 5:** Chewing simulation parameters

Parameter	Value
Vertical load	50 N
Frequency	1.61 Hz
Vertical movement distance	2 mm
Horizontal movement distance	0.7 mm
Vertical movement speed	11 mm/s
Horizontal movement speed	11 mm/s
Number of cycles	30,000 or 120,000

### Post-simulation digital scanning and volumetric wear analysis

After completion of the chewing-simulation protocol, all specimens were rescanned using the same scanner, positioning procedure, and acquisition settings used for the baseline measurements. The post-simulation stereolithography (STL) datasets were exported and paired with the corresponding baseline datasets for each specimen. Volumetric wear analysis was performed using Geomagic Design X software (3D Systems, Rock Hill, SC, USA). An initial alignment of the baseline and post-simulation STL datasets was performed using the reference notches on the acrylic specimen holders. An automated best-fit registration was then applied to the unchanged reference surfaces to standardize the three-dimensional superimposition. The acrylic holder and reference notches were excluded from the wear-measurement region. Following registration, the region of interest corresponding to the loaded specimen surface was segmented from the surrounding intact geometry. Volumetric material loss was calculated in cubic millimeters as the difference between the baseline and post-simulation volumes:

$$\Delta\mathrm{V}=\mathrm{V}\_\mathrm{baseline}-\mathrm{V}\_\mathrm{post-simulation}$$

The calculation was based on the paired surface geometries rather than on a color-deviation threshold. Accordingly, the color-deviation maps were used only for qualitative visualization of the location and distribution of surface changes and were not used to classify or calculate volumetric wear. Identical registration, segmentation, and calculation settings were applied to all specimens. The resulting volumetric material-loss values were recorded separately for each specimen and analyzed according to material, storage medium, and chewing-cycle number.

### Statistical analysis

Statistical analyses were performed using jamovi software (version 2.7.6; The jamovi Project, Sydney, Australia) with the Walrus module. The distribution of volumetric material-loss data was evaluated using the Shapiro-Wilk test and showed significant deviation from normality. Because the study had a factorial design and required evaluation of both main effects and interactions, a robust factorial analysis of variance based on 20% trimmed means was used. This approach reduces the influence of non-normality, heteroscedasticity, and extreme observations while retaining the factorial structure of the analysis [18].

A three-way robust ANOVA was conducted with material, storage medium, and chewing-cycle number as between-specimen factors. The model included the main effects of all three factors, all two-way interactions, and the three-way interaction. When appropriate, pairwise comparisons were performed using robust t-tests with Holm adjustment to control the family-wise Type I error rate.

The prespecified subgroup size of eight specimens, corresponding to a total of 128 specimens, was selected with reference to comparable in vitro chewing-simulation studies evaluating the wear behavior of occlusal splint materials [4–7]. No post hoc power analysis was reported because power calculated from the observed effect size provides limited additional information beyond the effect estimate and its confidence interval.

Descriptive results are presented as 20% trimmed mean ± standard error (SE). Effect sizes are reported as bootstrap partial eta squared (ηp²) with corresponding 95% confidence intervals (CIs). Statistical significance was set at *p* < 0.05.

## Results

### Volumetric wear analysis

All 128 specimens completed the experimental protocol and were included in the statistical analysis. No measurements were missing, and no observations were excluded solely on the basis of being identified as potential outliers.

Volumetric material loss was quantified by superimposing the baseline and post-simulation stereolithography (STL) datasets. Robust factorial ANOVA showed a significant main effect of material (*p* < 0.001, partial η² = 0.881). Chewing-cycle number also had a significant effect on volumetric material loss (*p* = 0.001, partial η² = 0.626), whereas the main effect of storage medium was not statistically significant (*p* = 0.054).

Among the interaction terms, only the material × chewing-cycle interaction was statistically significant (*p* = 0.001, partial η² = 0.752), indicating that the change in volumetric material loss with increasing cycle number differed among the tested materials. The material × storage-medium interaction (*p* = 0.474), storage-medium × chewing-cycle interaction (*p* = 0.370), and three-way interaction among material, storage medium, and chewing-cycle number (*p* = 0.128) were not statistically significant.

### Volumetric wear according to material type, storage medium, and chewing cycle

The condition-specific 20% trimmed means and corresponding standard errors are presented in Table 6. Across the experimental conditions, Cool&Flex generally showed the greatest volumetric material loss, whereas PEEK Biosolution and Armarez showed comparatively low values. Detax Freeprint Splint exhibited intermediate volumetric material loss.

**Table 6:** Volumetric material loss according to storage medium, chewing-cycle number, and material. Values are presented as 20% trimmed mean ± standard error (mm³); *n* = 8 specimens per experimental cell

Storage medium	Chewing cycles	Armarez PMMA	Detax Freeprint Splint	PEEK Biosolution	Cool&Flex
Gastric acid	120,000	0.128 ± 0.045	0.350 ± 0.112	0.060 ± 0.009	1.699 ± 0.395
Gastric acid	30,000	0.039 ± 0.018	0.165 ± 0.059	0.077 ± 0.024	0.453 ± 0.105
Artificial saliva	120,000	0.091 ± 0.034	0.513 ± 0.116	0.132 ± 0.041	2.298 ± 0.291
Artificial saliva	30,000	0.125 ± 0.061	0.310 ± 0.243	0.062 ± 0.023	0.540 ± 0.122

When the storage media and chewing-cycle conditions were pooled within each material, the trimmed mean volumetric material-loss values were 1.171 ± 0.193 mm³ for Cool&Flex, 0.296 ± 0.059 mm³ for Detax Freeprint Splint, 0.087 ± 0.020 mm³ for Armarez, and 0.079 ± 0.014 mm³ for PEEK Biosolution. These pooled estimates correspond to the overall material values reported in the Abstract and should be distinguished from the condition-specific estimates presented in Table 6.

Holm-adjusted pairwise comparisons showed that Cool&Flex had significantly greater volumetric material loss than the other tested materials (*p* < 0.05). Detax Freeprint Splint also showed significantly greater material loss than Armarez and PEEK Biosolution. No statistically significant difference was found between Armarez and PEEK Biosolution.

Volumetric material loss generally increased with the number of chewing cycles, although the magnitude of the increase differed among the materials, as indicated by the significant material × chewing-cycle interaction. Descriptive differences were observed between artificial saliva and simulated gastric acid; however, the main effect of storage medium and its interactions with the other experimental factors were not statistically significant. Among the factors examined, material showed the largest observed effect size, followed by chewing-cycle number.

### Three-dimensional wear pattern analysis

**Fig. 2:**
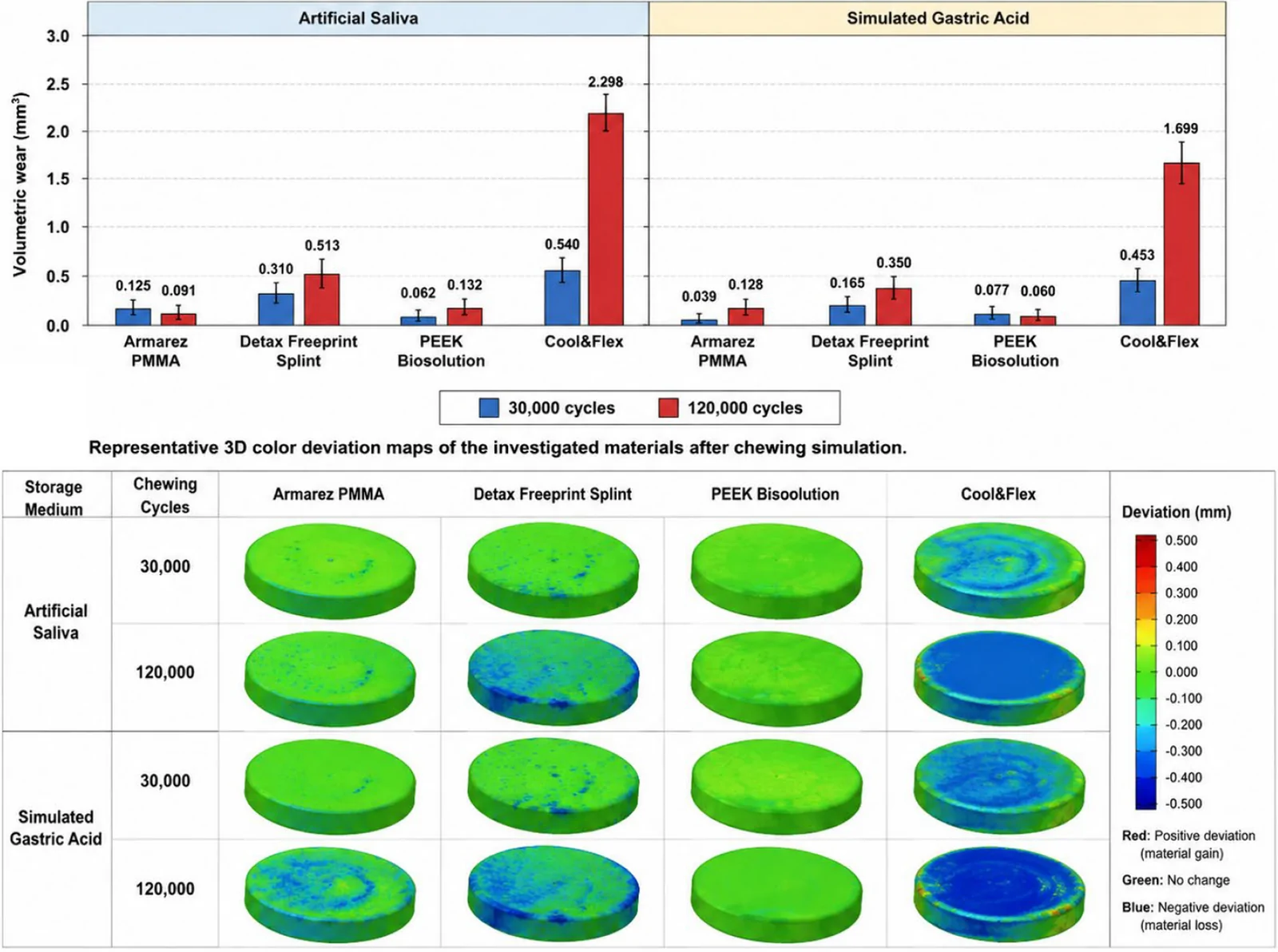
**A **Volumetric material loss (mm³) of the investigated occlusal splint materials after 30,000 and 120,000 chewing cycles in artificial saliva and simulated gastric acid. Error bars represent the standard error of the 20% trimmed mean. **B **Representative three-dimensional color-deviation maps obtained after chewing simulation. Blue regions indicate negative surface deviation relative to the baseline scan, green indicates minimal dimensional change, and warm colors indicate positive surface deviation. The deviation maps are provided for qualitative visualization

The combined associations of material, storage medium, and chewing-cycle number with volumetric material loss are illustrated in Fig. 2.

The graphical presentation in Fig. 2A shows the condition-specific volumetric material-loss values for the tested materials. The increase associated with the higher cycle number was most pronounced for Cool&Flex. PEEK Biosolution and Armarez generally maintained comparatively low values, whereas Detax Freeprint Splint showed intermediate material loss.

The representative three-dimensional color-deviation maps in Fig. 2B were consistent with the quantitative findings. The most extensive surface alterations were observed in Cool&Flex specimens after 120,000 chewing cycles, whereas comparatively limited geometric changes were observed in PEEK Biosolution and Armarez specimens. Detax Freeprint Splint showed an intermediate pattern. Across the experimental groups, the observed surface changes were predominantly localized within the antagonist contact region.

The color-deviation maps were used for qualitative visualization only and were not included as independent quantitative outcome measurements. 

**Fig. 3:**
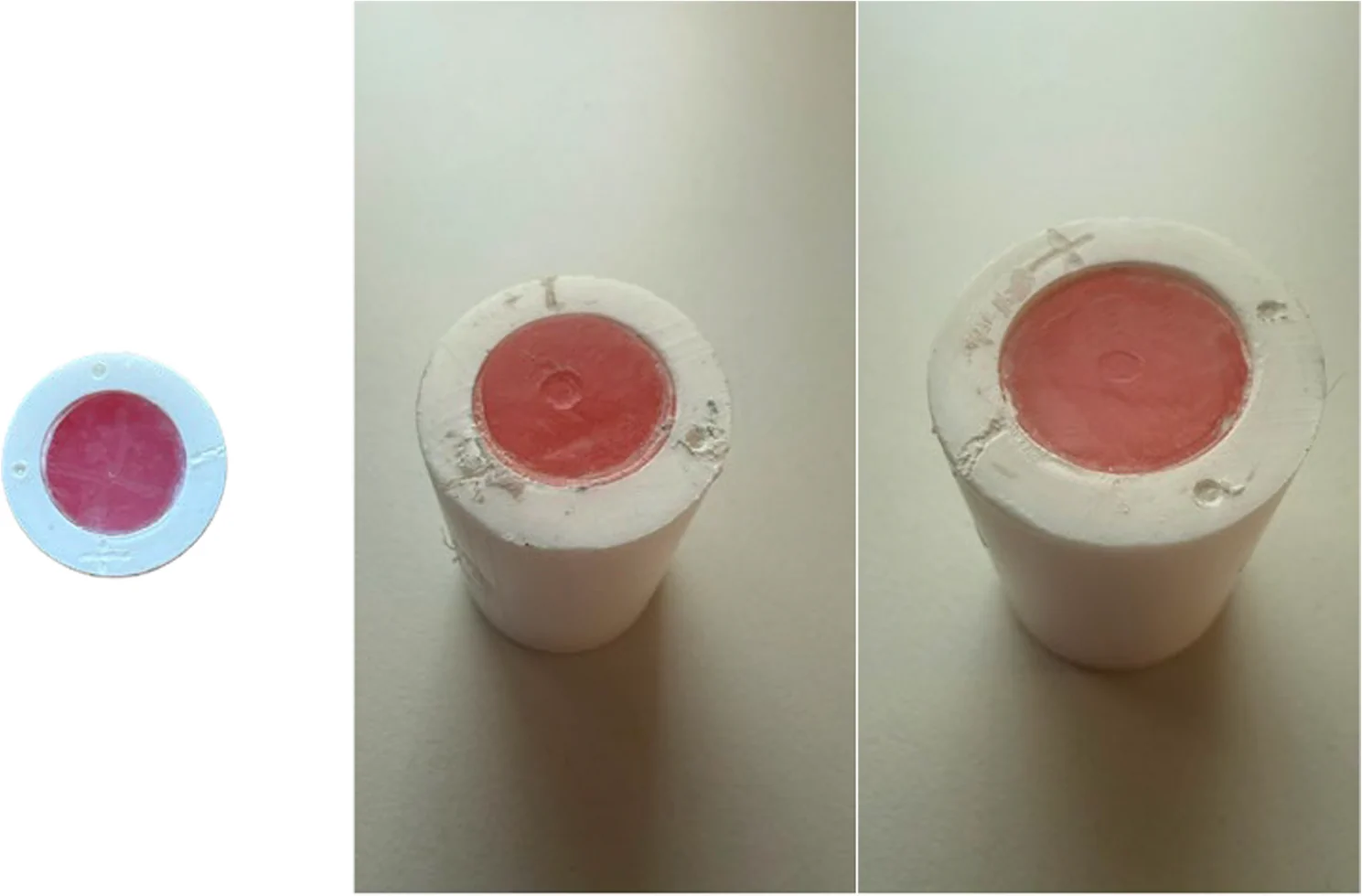
Representative macroscopic photographs of the investigated occlusal splint materials before and after chewing simulation. The images are provided for qualitative illustration only; quantitative volumetric material loss was determined through three-dimensional superimposition of the baseline and post-simulation STL datasets

Representative macroscopic photographs of the specimens before and after chewing simulation are presented in Fig. 3. Comparatively limited visible surface changes were observed in the PEEK Biosolution and Armarez specimens, whereas more distinct wear facets were observed in Detax Freeprint Splint and particularly in Cool&Flex after 120,000 chewing cycles. These qualitative observations were generally consistent with the volumetric material-loss measurements.

## Discussion

The present in vitro study evaluated the volumetric wear behavior of four occlusal splint materials after chewing simulation in artificial saliva and simulated gastric acid. The null hypothesis was partially rejected because material and chewing-cycle number significantly affected volumetric material loss, whereas storage medium did not have a statistically significant main effect. The significant material × chewing-cycle interaction further indicated that the response to increased mechanical loading differed among the tested materials.

Among the investigated factors, material showed the largest observed effect size. Previous studies have similarly reported differences in wear behavior among conventionally processed, CAD/CAM-milled, and additively manufactured occlusal splint materials [5–8]. Recent investigations have also indicated that wear performance may depend on the combined influence of material composition, product-specific formulation, processing conditions, and manufacturing method rather than on fabrication category alone [15–17]. In the present study, the two DLP-printed PMMA-based products exhibited different volumetric material-loss values despite being produced using the same general manufacturing technology. This finding suggests that product-specific formulation and processing characteristics may contribute to wear behavior. However, chemical composition, polymer architecture, degree of conversion, and related material properties were not directly measured in this study; therefore, their individual contributions cannot be determined from the present findings.

PEEK Biosolution exhibited comparatively low volumetric material loss across the experimental conditions. This finding may be related to the mechanical and chemical characteristics previously reported for PEEK-based dental materials, including its semi-crystalline structure, favorable mechanical behavior, and low water sorption [9–11]. Its production as an industrially processed CAD/CAM block may also contribute to structural homogeneity. Previous studies have reported favorable wear behavior for industrially processed CAD/CAM polymers compared with some conventionally processed or additively manufactured materials [11, 16]. Nevertheless, these possible mechanisms were not evaluated directly in the present study, and the observed performance should be attributed specifically to the tested PEEK Biosolution product. Moreover, differences in materials, antagonists, loading protocols, and measurement methods limit direct quantitative comparisons across studies [15].

Armarez also showed comparatively low volumetric material loss and did not differ significantly from PEEK Biosolution. Because Armarez is a DLP-printed PMMA-based resin rather than an industrially polymerized CAD/CAM block, its performance cannot be explained solely by the manufacturing category. Its behavior may be associated with product-specific factors such as resin formulation, polymer-network characteristics, printing parameters, and post-curing response. These properties were not independently assessed, and a causal explanation cannot therefore be established. However, the result is consistent with studies showing considerable variation among commercially available additively manufactured occlusal-device materials [15, 16].

The difference between Armarez and Detax Freeprint Splint further indicates that products manufactured using the same additive technology should not be assumed to have equivalent wear behavior. Both were categorized as DLP-printed PMMA-based materials and were produced using standardized procedures, but Detax Freeprint Splint showed greater volumetric material loss. Differences in resin composition, polymerization behavior, interlayer bonding, and response to post-curing may have contributed to this result. Because these characteristics were not measured, this interpretation remains explanatory rather than confirmatory. The findings nevertheless support evaluating additively manufactured materials at the individual product level rather than generalizing performance across the entire category [15, 16].

Cool&Flex exhibited greater volumetric material loss than the other tested products. Polyamide materials are generally characterized by flexibility and impact resistance, but their lower stiffness and surface hardness relative to some high-performance engineering polymers may increase susceptibility to deformation and material displacement during repeated loading. Moisture uptake associated with polar amide groups may also affect the mechanical behavior of the polymer matrix. These mechanisms could partly explain the observed findings but were not directly investigated. Previous studies evaluating flexible polymers and occlusal splint materials have similarly reported that polymer composition and processing method may affect wear resistance [5–8, 17]. However, methodological differences between studies, including antagonist selection, loading protocol, environmental conditions, and wear-assessment technique, should be considered when comparing results.

Detax Freeprint Splint demonstrated intermediate wear behavior, with less volumetric material loss than Cool&Flex but greater loss than Armarez and PEEK Biosolution. Its difference from Armarez cannot be attributed solely to additive manufacturing because both products were fabricated using DLP technology. Variables such as printing orientation, layer thickness, exposure settings, cleaning, interlayer bonding, and post-curing can influence the mechanical behavior of printed polymers. In the present study, printing orientation and nominal layer thickness were standardized. Consequently, the observed difference may reflect an interaction between product-specific material characteristics and the standardized processing protocol, although these mechanisms require direct investigation.

Increasing the number of chewing cycles significantly increased volumetric material loss, and the magnitude of this increase differed among the tested materials. The largest increase was observed for Cool&Flex, whereas PEEK Biosolution and Armarez maintained comparatively low values at both cycle levels. Detax Freeprint Splint showed an intermediate response. The use of two cycle protocols therefore provided information about the response of each product to different levels of simulated mechanical loading. Nevertheless, the cycle protocols should not be regarded as direct predictions of clinical service because intraoral wear is affected by numerous patient- and treatment-related variables.

Storage medium did not have a statistically significant main effect on volumetric material loss. Similarly, the material × storage-medium, storage-medium × chewing-cycle, and three-way interactions were not statistically significant. These results indicate that simulated gastric acid did not produce a detectable increase in volumetric material loss under the conditions evaluated. However, the absence of statistical significance should not be interpreted as evidence that acidic exposure has no effect on dental polymers. Previous studies have shown that simulated gastric acid may affect surface hardness, roughness, flexural properties, and surface integrity depending on the tested material and exposure protocol [9, 10]. Such surface changes may not necessarily produce a measurable change in volumetric material loss over the experimental period used here.

The continuous immersion model also differs from clinical gastric acid exposure, which is generally intermittent and is followed by salivary clearance and buffering. The oral environment additionally involves temperature changes, enzymes, biofilm, dietary factors, and variations in loading. Therefore, the present findings regarding simulated gastric acid should be restricted to the experimental conditions evaluated and should not be extrapolated directly to the long-term intraoral performance of splints used by patients with gastroesophageal reflux disease.

The three-dimensional color-deviation maps were generally consistent with the quantitative volumetric findings. Surface changes were predominantly localized within the antagonist contact region. Cool&Flex showed the most extensive geometric alteration, particularly after 120,000 cycles, whereas PEEK Biosolution and Armarez showed comparatively limited changes. Because the deviation maps were used for qualitative visualization, the volumetric measurements remain the primary basis for comparison.

From a clinical perspective, the findings suggest that product-specific wear behavior may be relevant when selecting an occlusal splint material. PEEK Biosolution and Armarez showed comparatively low volumetric material loss under the tested conditions, whereas Cool&Flex showed greater loss. However, the study did not evaluate complete splints, patient-related variables, treatment outcomes, or clinical longevity. Accordingly, the findings should not be used as direct evidence of the superiority of one product for a particular patient group. Long-term clinical studies are required before material-specific recommendations can be made.

The results should be interpreted within the limitations of the in vitro design. Standardized disc-shaped specimens do not reproduce the geometry, thickness variation, occlusal contacts, or stress distribution of clinical occlusal splints. The stainless-steel antagonist provided standardized contact geometry but differed from natural enamel and restorative materials. Thermocycling was not performed, and the specimens were not exposed to salivary enzymes, biofilm, dietary components, pH fluctuations, or intermittent acidic challenges. Continuous immersion in a single assigned medium also differs from clinical exposure patterns.

Volumetric material loss was evaluated without simultaneous assessment of surface roughness, hardness, microscopic damage, mass change, degree of conversion, or water sorption. Consequently, the mechanisms underlying the differences among the products cannot be determined directly. Not all machine-specific processing parameters for the milled and injection-molded products were quantitatively recorded. In addition, only four commercial products were examined; therefore, the results should not be generalized to all PEEK, PMMA-based photopolymer, or polyamide materials. Methodological characteristics and principal findings of representative previous studies are summarized in Table 7. 

**Table 7:** Comparison of previous studies evaluating occlusal splint materials and the present study

Study	Materials and manufacturing technique	Experimental protocol	Evaluation method	Major findings	Contribution relative to the present study
Brandt et al. (2019) [14]	Laboratory-made and CAD/CAM occlusal devices	Clinical evaluation	Clinical assessment	CAD/CAM-fabricated splints showed satisfactory clinical performance.	Clinical investigation; no quantitative laboratory wear analysis or comparison of different printable materials.
Berli et al. (2020) [12]	Pressed, milled, and 3D-printed occlusal device resins	Mechanical testing	Flexural and mechanical property evaluation	Mechanical properties differed according to fabrication technique.	Wear resistance and volumetric material loss were not investigated.
Wesemann et al. (2021) [11]	Injection-molded, milled, and additively manufactured occlusal splints	Accuracy and fit assessment	Accuracy and fit measurements	Manufacturing technique significantly affected splint accuracy and fit.	No chewing simulation or wear evaluation was performed.
Gibreel et al. (2021) [13]	CAD/CAM and conventional splint materials	Material characterization	Mechanical characterization	CAD/CAM materials generally demonstrated superior mechanical properties.	Did not evaluate wear behavior under simulated mastication.
Lawson et al. (2025) [15]	Commercial DLP 3D-printed occlusal device materials	Simulated wear testing	Wear resistance assessment	Significant differences were observed among commercially available printable resins.	Included only 3D-printed materials; no comparison with CAD/CAM PEEK, polyamide, or gastric acid exposure.
Aminuddin and Petridis (2026) [16]	Conventional, milled, and 3D-printed occlusal splint materials	Flexural strength, monomer release, and wear testing	Mechanical and wear evaluation	Material formulation and manufacturing technique both influenced wear performance.	Did not compare different commercial DLP PMMA resins under identical processing conditions or evaluate acidic aging.
Arreaza (2024) [17]	Occlusal splint materials fabricated using different processes	Wear testing	Wear evaluation	Polymer composition and fabrication process significantly influenced wear behavior.	Master’s thesis; combined chemical and mechanical aging was not evaluated.

## Conclusions

Within the limitations of this in vitro study, volumetric material loss differed significantly among the four commercial occlusal splint materials tested. PEEK Biosolution and Armarez showed comparatively low volumetric material loss, Detax Freeprint Splint showed intermediate loss, and Cool&Flex showed the greatest loss under the evaluated conditions.

A higher number of chewing cycles was generally associated with increased volumetric material loss; however, the magnitude of this change differed among the tested materials. Storage medium did not have a statistically significant main effect, indicating that simulated gastric acid did not produce a detectable increase in volumetric material loss under the conditions of this experiment.

Because polymer composition and manufacturing method varied simultaneously among the commercial products, their independent effects cannot be distinguished. The findings are therefore specific to the products and experimental conditions evaluated and should not be interpreted as direct predictions of clinical longevity. Further studies using clinically shaped splints and long-term clinical designs are required to determine the relevance of these differences to appliance performance.

## Data Availability

The datasets generated and analyzed during the current study are available from the corresponding author upon reasonable request.

## Abbreviations

3D: Three-dimensional
CAD/CAM: Computer-aided design/computer-aided manufacturing
CI: Confidence interval
DLP: Digital light processing
GERD: Gastroesophageal reflux disease
PEEK: Polyetheretherketone
PMMA: Polymethyl methacrylate
SE: Standard error
STL: Standard tessellation language
TMD: Temporomandibular disorder
